# Colour Me Blue: The History and the Biotechnological Potential of Pyocyanin

**DOI:** 10.3390/molecules26040927

**Published:** 2021-02-10

**Authors:** Thiago Gonçalves, Ulrich Vasconcelos

**Affiliations:** Centro de Biotecnologia, Departamento de Biotecnologia, Universidade Federal da Paraíba, R. Ipê Amarelo, s/n, Campus I, João Pessoa PB-CEP 58051-900, Brazil; thigoca@gmail.com

**Keywords:** bacterial pigments, *Pseudomonas aeruginosa*, pyocyanase, bioprospecting

## Abstract

Pyocyanin was the first natural phenazine described. The molecule is synthesized by about 95% of the strains of *Pseudomonas aeruginosa.* From discovery up to now, pyocyanin has been characterised by a very rich and avant-garde history, which includes its use in antimicrobial therapy, even before the discovery of penicillin opened the era of antibiotic therapy, as well as its use in electric current generation. Exhibiting an exuberant blue colour and being easy to obtain, this pigment is the subject of the present review, aiming to narrate its history as well as to unveil its mechanisms and suggest new horizons for applications in different areas of engineering, biology and biotechnology.

## 1. Introduction

Secondary metabolites are essential requirements for promoting the maintenance and persistence of producing organisms [1,2]. Bacterial pigments are secondary metabolites with varied colours, endowed with a heterogeneous molecular structure and low molecular weight [3]. A large number of bacteria produce pigments which are assigned different functions, such as photosynthesis [4], protection against ultraviolet radiation [5], iron uptake [6], ecological interactions with other organisms [7] and participation in the signalling that modulates gene expressions dependent on cell density [8].

Phenazines are molecules with heterocyclic rings containing nitrogen, which have different physical and chemical properties based on the type and position of the functional groups present [9]. In addition, phenazines are synthesized by a limited number of *Bacteria* and *Archaea*. At least 10 types of phenazine occur simultaneously in a single organism, however more than 50 naturally occurring phenazine have been described, with an emphasis on pyocyanin, considered the most important compound in the group [10].

Pyocyanin is an exuberant blue-coloured phenazine, produced exclusively by 90–95% of *Pseudomonas aeruginosa* strains [11]. Given the physical-chemical and biological properties of pyocyanin, the molecule has great potential to be applied to different areas of biology, engineering and biotechnology. The pigment exhibits several advantages, namely: (1) it is natural, biodegradable and environmentally-friendly; (2) the producing organism is easy to manipulate and selects the strains with the highest yield, using simple means of cultivation with low cost substrates; (3) it may be used to produce biomass and; (4) the collection and extraction processes are quick and simple compared to any other chemical synthesis process [12,13]. This work presents some considerations about the history, chemistry, collection and applications of pyocyanin.

## 2. History and Chronology of Studies with Pyocyanin

The pigment was first described in 1860 by Fordos, when observing a bluish purulent sample, from an infection caused by *P. aeruginosa*. The name of the pigment was proposed by the combination of Greek words, used to designate pus and the colour blue [14]. Dr. Fordos also described different properties of the pigment, such as its solubility and the colours it exhibited at different pH levels. He proposed that the bacterium exhibits four different types of colours. This was later known as the chameleon effect [15].

The first isolation of the pigment from the “pyocyanic bacillus” occurred in 1882 when Gessard attempted to verify the parasitic origin of the phenomenon that gave the colour blue to pus and tissues close to an infection [16]. In addition, Jordan in 1899 identified pyocyanin spread throughout a common laboratory culture medium [17]. The pure compound was isolated only in 1924, becoming the first natural phenazine obtained and purified in a laboratory [18]. In 1929, Wrede and Straek proposed the chemical structure of pyocyanin, later corrected by Hillemann, in 1938 [19].

Waksman, in his last published manuscript, presented a retrospective on antibiotic therapy and reminded the reader that between late 1941 and early 1942, he coined the word “antibiotic”, in response to a request to create a word to designate compounds and preparations with a defined chemical structure, which produced a therapeutic effect against infectious diseases [20]. In this work, he cited pyocyanase as one of those compounds. Pyocyanase was the first formulation to use the potential of pyocyanin in therapy [21].

The term antibiosis was coined by Vuillemin in 1889 to designate the natural selection of one organism over another. Ten years later, Ward extended the term to define microbial antagonism. At the same time, it was understood that not only the presence of certain microbes prevented the growth of others, but that the phenomenon also occurred due to the action of substances produced by these organisms [22].

In the same year, Emmerich and Löw isolated pyocyanase from a macerate of *P. aeruginosa* cultures. The lysate was not initially identified as a molecule, being erroneously described as an enzyme mixture, reflected in the nomenclature used to designate the compound. Scientists also observed that the pyocyanase produced by the “pyocyanic bacillus” could be used to treat diphtheria and against meningococci. In addition, it served as a mouthwash and years later it was shown to be effective against anthrax. Pyocyanase in the form of eye drops, sprays and mouthwashes were the most common presentations because the systemic use proved to be very toxic. Thus, pyocyanase was probably the first antibiotic produced industrially and applied therapeutically in humans, decades before the discovery of penicillin and the era of antimicrobial chemotherapy [23,24].

In the early years of interest in this pigment, encouraged by the attraction for the colour, the most investigated function was its antimicrobial activity. In the early 20th century, pseudomonads were the largest group of non-differentiating microbes used to produce antibiotics. Three compounds reached the stage of clinical application: pyocyanin acids, pyrrolnitrin and pyocyanase [25]. With the advancement of research, pyocyanase demonstrated protection of experimental animals against rabies and the vaccinia viruses. From the beginning, however, the compound was identified as more effective against Gram-positive bacteria than Gram-negative bacteria and other organisms [26].

A hydroalcoholic solution containing 10,000 to 20,000 units/mg of pyocyanase was seen to provide antimicrobial activity when diluted 1:10 and 1:20. Antimicrobial activity was reported against staphylococci, streptococci, pneumococci, gonococci and *V. cholerae*, however, there was no consensus on the active principle of the lysate, given the fact that pyocyanase was not an enzyme. Between 1909 and 1928, much controversy was raised regarding the use of pyocyanase. The aqueous solution was unstable and lasted only a week [22]. On the other hand, the cell suspension or the lipid extract obtained from the cultures, containing a suspension of a crystalline material, appeared to be more effective, leading scientists to deduce that the active substance of pyocyanase was the lipids derived from *P. aeruginosa*. The clinical use of pyocyanase however became secondary due to significative side effects reported, including severe damage to tissues and mucosa [27]. In 1935 Kramer discovered that not all the strains of *P. aeruginosa* produced pyocyanase or lost it, for some unrevealed reason [28].

Over the passage of time, pyocyanin fell into disuse. The 1950s, however, marked a decade of studies interested in the development of culture media for obtaining the pigment. The production of pyocyanin by traditional means, as commonly used in the routine of a microbiology laboratory, is based on the energetic state of the bacterium. This is reduced to a low concentration of nutrients, resulting in decreased growth rate and increased pigment concentration [29]. Nutritional scarcity, especially related to PO_4_^−3^ and Ca^+2^, forces the pyocyanin strains to develop the pigment that propagates through the medium [30]. This production generally starts at the beginning of the stationary phase, which is dependent on the generating time of the strain. The bacterium under these cultivation conditions tends to exhibit a generation time ranging between 3–6 h [31], resulting in the blue-green colour that appears between 48–72 h after the beginning of the incubation.

Chorismic acid as a precursor molecule of pyocyanin was discovered in the 1960s, a decade dedicated to understanding the biochemical pathways of *P. aeruginosa* in pigment synthesis. The following two decades explored the physiological role of pyocyanin for the bacterium. The mechanisms of action of pyocyanin began to be understood, opening up old questions and revealing the metabolic and ecological advantages of *P. aeruginosa*, compared to other organisms [32]. Competition is a natural process and occurs when one organism produces a substance with an inhibitory effect on the growth of another; this relationship ensures the balance of species in coexistence, as well as the entire ecosystem [33]. These substances may be of various natures, such as pigments, enzymes, organic acids and antibiotics. In addition, variations in temperature, pH, nutrient and oxygen levels, as well as population concentration are extrinsic factors that influence the pyocyanin synthesis. In this context, *P. aeruginosa* naturally has an advantage over other microbes [34,35,36,37].

Between the 1990s and 2010s, the focus of studies involving pyocyanin tried to elucidate the genetic, molecular and biochemical basis of the regulation of phenazine synthesis, including pyocyanin [37,38,39]. It was observed that the locus responsible for phenazine biosynthesis is highly conserved in *Pseudomonas* spp. The production varies according to the specie and is strongly influenced by nutritional factors or genes. The expression of their regulatory systems is dependent on temperature [40]. From this epoch on, pyocyanin gained new status and different applications were tested and proposed, as discussed below.

## 3. *Pseudomonas aeruginosa*: The Exclusive Natural Pyocyanin Producer

*Pseudomonas aeruginosa* is a ubiquitous Gram-negative rod preferring a saprophyte terrestrial and aquatic environment. *P. aeruginosa* is the main representative of fluorescent pseudomonads, the most diverse group of *Pseudomonas* ssp. fluorescent pseudomonads are composed of more than 140 species, which are constantly being reclassified, given the biodiversity of the genus *Pseudomonas* [41,42]. The bacterium has a polar flagellum and measures about 0.5–1.0 μm wide by 1.0–1.5 μm long [43]. Its remarkable metabolic versatility guarantees greater resistance and tolerance of *P. aeruginosa* to a myriad of compounds, confirming its reputation as resilient in environments with different degrees of selective pressures and oxidative stresses [44,45].

The *P. aeruginosa* genome measures a considerable 6.3 Mpb (6,264,403 bp). To it may be attributed, among other characteristics, phenotypic diversity and metabolic versatility, reflected in the ability to use more than 90 molecules as sources of carbon and energy [46,47]. In addition, the bacterium is not a fastidious microbe and can grow in simple culture media and low-cost substrates. When cultivated in a solid medium, the colonies have an average diameter between 0.8 and 2.2 μm and take on a greenish colour due to the synthesis of two main pigments produced, blue (pyocyanin) and yellow (fluorescein). Another characteristic of laboratory cultivation is its fruity aroma of grapes, derived from the synthesis of 2-aminoacetophenone [48,49].

The bacterium is not considered an obligate pathogen, but a primarily opportunistic pathogen. The species exhibits different phenotypes associated with virulence factors, such as alginate production, adhesins, neuraminidases, lipopolysaccharide, exotoxin A, enterotoxin, exoenzyme S, phospholipase C, elastase, leukocycline and pigments [50,51]. In addition, *P. aeruginosa* may synthesize at least six different pigments: fluorescein [52], pyoverdine [53], pyomelanin [54], aeruginosin A, aeruginosin B [55] and pyocyanin [7]. The last three listed pigments are phenazines.

The production of pigments, especially pyocyanin, enhances the expression of virulence factors and other phenotypes that converge on *P. aeruginosa*. In addition, antibiotic resistance does not appear to be associated with pyocyanin production. The differences in the resistance profile demonstrate that *P. aeruginosa* applies simultaneous multifactorial mechanisms. The strains that exhibit pyocyanin however, have a prevalence of higher multidrug-resistance, as well as more virulence factors, compared to non-producing strains [56].

Phenazine-producing organisms exhibit an increase in life-span in any environment in which they develop. This is extremely relevant for maintenance in the environment [57]. *P. aeruginosa* can grow in oligotrophic environments and produces less pyocyanin when exposed to water, compared to enriched media or soil [58]. The preferred lifestyle of the bacteria is in biofilm [59]. Biofilms are sessile microbial communities in which cells live in a complex closed association network, surrounded by an adhesive polymeric matrix (EPS) [60]. The carbon source and nutrient levels are the main factors that govern the establishment of a biofilm [61]. The reduction of nutrients to produce EPS obliges the bacteria to maintain a planktonic lifestyle [62].

The current model of architecture and organization of microbial biofilms is based on the *P. aeruginosa* lifestyle. The biofilm exhibits a complex three-dimensional dynamic structure composed of numerous microenvironments, characterized by different gradients [63]. This makes it possible to establish a variety of phenotypes in a single biofilm [64]. In addition, the architecture is dependent on the type of available carbon present in the medium, taking the form of a pillar in the presence of citrate, or a mushroom in the presence of glucose [65,66].

## 4. Pyocyanin: Fundamentals and General Properties of the Molecule

Pyocyanin is a compound soluble in chloroform and diffusible in water, produced at an optimum pH ranging from 7.4 to 8.4, but not less than 6.0 or greater than 9.0 [15]. The most striking property of this metabolite is its intense blue colour, however the absorption characteristics in the spectrum are pH dependent. In the oxidized form, the compound exhibits absorption bands with λ_max_ between 230 and 380 nm and one second, lower, around 700 nm, related to the protonated and deprotonated forms of the pigment [46,67].

Pyocyanin is a zwitterion with a molecular weight of 210.23 g/mol [68,69]. Since it is a phenazine, the core structure of the molecule is a pyrazine ring (1,4-diazabenzene). Pyocyanin is composed of two subunits of N-methyl-1-hydroxyphenazine and easily crosses biological membranes, serving as an electron carrier for *P. aeruginosa*. Preferably, the pigment donates an electron to molecular oxygen, resulting in a blue colour. In addition, the greatest role of pyocyanin in *P. aeruginosa* occurs under anaerobiosis or microaerophilia. In these cases, pyocyanin accepts electrons generated from NADH during the oxidation of assimilated carbon sources [26].

The phenol group attributes an acidic characteristic to pyocyanin, pKa = 4.9 [68]. The molecule however has three states: ionized at physiological pH (blue colour), protonated in an acidic environment (red colour) and neutral (blue colour). Thus, pyocyanin can cross the membrane and penetrate cells, assuming a multifunctional role for *P. aeruginosa* [70]. In addition, the bacteria can also mediate a cycle of autooxidation of pyocyanin. In this case, two electrons are transferred to the oxidized form, generating the reduced form, which can be converted back to its oxidized form by molecular oxygen. During the autooxidation cycle, reactive oxygen species (ROS) may also be generated [71].

**Figure 1:**
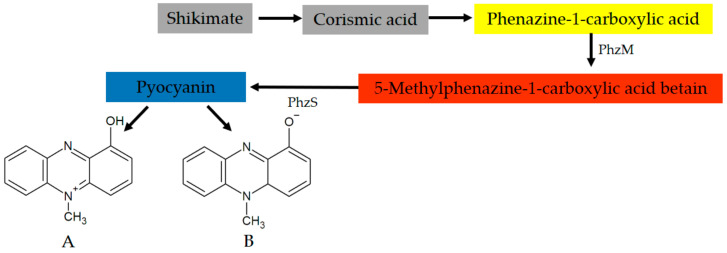
Steps of the biosynthesis of pyocyanin by *Pseudomonas aeruginosa* and its oxidated (**A**) and reduced (**B**) states.

The precursor molecule of pyocyanin is chorismic acid (CA), the end product on the shikimate pathway [72]. The conversion of CA to phenazine-1-carboxylic acid (PCA) is controlled by seven genes, encoded by two operons (*phzA1B1C1D1E1F1G1* and *phzA2B2C2D2E2F2G2*). Then, the synthesis of pyocyanin appears two steps from the PCA (Figure 1). These steps are regulated by two main genes, *phzM* and *phzS* [26]. PCA is converted to 5-methylphenazine-1-carboxylic acid betaine (MPCBA), by means of a phenazine-specific methyltransferase (PhzM). In the second step, MPCBA is catalysed by flavin-dependent monooxygenase (PhzS), involving the hydroxylation of the MPCBA betaine to 1-hydroxy-5-methyl phenazine, i.e., pyocyanin [11,73].

It is noteworthy that PCA is an active phenazine in *P. aeruginosa* [34]. In addition, other intermediate phenazines produced by the bacterium also exhibit antimicrobial activity [74]. The result implies that these molecules can offer ecological benefits similar to those attributed to pyocyanin in non-producing strains of *P. aeruginosa* [75]. In addition, PhzM is only active in the presence of PhzS, suggesting that a protein-protein interaction is involved in the formation of pyocyanin. PhzS displays disorder near the binding site and this can be part of the substrate recognition process, which allows PhzM to form the PhzM-PhzS complex [76]. In addition, PhzM has no activity on PCA. Pyocyanin is only produced when PhzS and NADH are present because a transient physical interaction is required to activate pyocyanin production [77].

## 5. Modulation and Regulation of Pyocyanin Production

Pyocyanin is predominantly produced by edaphic strains of *P. aeruginosa*, more than in any other environment in which it develops. Interestingly, there are no geographical differences between the producing strains [78]. The phenazine-producing microbes are dominant, compared to the non-producing organisms. Phenazine production is virtually a requirement for ecological competence and persistence of soil microorganisms, including in the presence of allochthonous microbiota [79].

The synthesis of pyocyanin is modulated by a quorum-sensing (QS) mechanism that has been widely studied in *P. aeruginosa* [80]. The QS is a mechanism dependent on information from the bacterial population delivered by small diffusible molecules, called autoinducers, produced by each cell individually, regardless of the concentration of nutrients in the medium and secreted at basal level from a minimum of 500 cells [59,81].

The main autoinducers of *P. aeruginosa* are acyl-homoserine lactone (AHL) and Pseudomonas quinolone signal (PQS). Both are involved in the expression and modulation of pyocyanin [82]. The increase in AHL concentration results in an increase in the production of pyocyanin by the bacterium [83]. Additionally, *P. aeruginosa* has two main QS systems, extensively studied: LasR-LasI and RhlR-RhlI systems. They upregulate different bacterial expressions, such as alginate, rhamnolipid and pyocyanin synthesis. Negative control for the case of pyocyanin expression is exercised by the *PtsP* gene that interacts with the two QS systems, in addition to a repressor, QscR. This repressor encodes the *qscR* gene, responsible for the downregulation of QS systems in *P. aeruginosa* [84,85].

Pyocyanin also integrates the QS network indirectly by increasing the expression of the MvaT and MvaU proteins, related to the regulation of the PQS system. When the levels of these proteins are increased, pyocyanin production increases, because the PQS system is stimulated. These same proteins are involved in the QS responses to the formation of biofilm in *P. aeruginosa* [86], indirectly recognizing pyocyanin as a crucial element in the events associated with the establishment of biofilm by the bacterium [87].

Environmental conditions also influence the production of pyocyanin, particularly pH, temperature, oxygen tension and oxidative stress. The availability of nutrients however governs production more significantly. Deficiency of Mg and some important ions, such as Fe^3+^, SO_4_^2−^, PO_4_^2−^ and NH_4_^+^, also plays a crucial role [88]. In addition, the presence of glycerol and TCA intermediates are required as a carbon source, as well as peptone and certain amino acids as nitrogen sources to build the phenazine ring. Additionally, a curious characteristic of *P. aeruginosa* is its expression of pyocyanin via AHL produced by coexisting microbes [89].

Suppression of pyocyanin synthesis may also occur in response to the availability of preferential carbon sources. *P. aeruginosa* prefers TCA intermediates, for example, succinate, because the compound interacts with PhzM mRNA, which encodes the key enzyme to produce pyocyanin. In addition, pyocyanin production appears to require excretion of pyruvate to reduce the intracellular NADH/NAD^+^ ratio, suggesting that the *P. aeruginosa* regulates primary metabolism during the exponential phase and the primary metabolism regulates pyocyanin production. In short, the NADH/NAD^+^ ratio is balanced by the inactivation of the pyruvate dehydrogenase complex by pyocyanin. The reduction of NAD^+^ can be avoided because pyruvate may be excreted without further oxidation [90].

The pigment is responsible for pyruvate secretion also in the stationary phase, influencing the intracellular redox state by reducing the flow of carbon through the central metabolism pathways. The reaction with NADH may represent an adaptation of the bacteria to modulate its intracellular redox state. Homeostasis is regulated after reducing pyocyanin when oxygen is available, resulting in increased aerobic respiration and NADH reoxidation. The reduction of pyocyanin also provides redox balance in the absence of electron acceptors. With a limited amount of electron acceptors, there are two types of NADH reoxidation, either with a reduction of pyocyanin directly or mediated by phenazine reductase [91].

In addition, protein degradation products under the action of alkaline protease are used to produce pyocyanin. *P. aeruginosa* also regulates the pigment concentration by means of an autoinducer secreted in accumulated basal concentrations; alkaline protease, however, is not mandatory for this [92]. In addition, nitrous oxide (NO) also exerts activity in the pyocyanin biosynthesis. Accumulation of NO induces a reduced pigment production. This is because NO strongly suppresses the *nor* gene, which encodes NO reductase, an enzyme that participates in the NO reduction reaction to N_2_O [93].

## 6. Mechanism of Action of Pyocyanin

*P. aeruginosa* was the first microbe studied in terms of the ability to inhibit other organisms [94]. Pyocyanin acts by causing oxidative stress in susceptible prokaryotes and eukaryote cells, through the flow of electrons and the accumulation of ROS, especially O^2−^ and H_2_O_2_, after reaction with molecular oxygen [11,32]. The lethal concentration of pyocyanin against bacteria, filamentous fungi, yeasts, protozoa, algae and small animals varies significantly from study to study, depending on the model organism evaluated, and may occur from minute concentrations up to about 2000 µg/mL [95,96]. In Table 1, some organisms susceptible to the action of pyocyanin are summarized. The virulence mechanism used by *P. aeruginosa* is evolutionarily conserved and applied to different susceptible organisms [97].

As a planar molecule with hydrophobic and hydrophilic properties, pyocyanin interacts easily with the membrane of several organisms [98]. The formation of intracellular ROS in host cells after exposure to pyocyanin results in oxidative damage to the components of the cell cycle, depletion of NAD(P)H and enzymatic inhibition [68], in addition to specific damage to DNA [99]. The reduction of NAD(P)H and subsequent generation of ROS is irreversible possibly involving ring cleavage of the pyocyanin skeleton; however, pyocyanin can be oxidized by H_2_O_2_, formed in the oxidation of NAD(P)H by pyocyanin itself and catalysed by microperoxidases. This may be a relevant strategy for *P. aeruginosa* in in vivo conditions [100].

Initial investigations have already shown that oxygen is essential for the activity of pyocyanin against its competitors, and that the bactericidal effect depends on the concentration of the pigment. This may result in reductions ranging from one to 8 log unit cells/mL of the sensitive organism [101].

In eukaryotes, on the other hand, the interaction of pyocyanin can occur at the level of the cell wall or membrane, as well as in the respiratory chain of the mitochondria [102]. This interaction results in the release of mitochondrial ROS, accelerating the process of senescence and apoptosis [103]. In addition, concentrations less than 5 µg/mL of the pigment can disturb the vegetative state of certain filamentous fungi, promoting significant inhibition of the growth of the vegetative mycelium and the development of reproductive mycelia [10].

The formation of O^2−^ is the primary mechanism of the antimicrobial effect of pyocyanin. The ion interacts with the membrane, resulting in inhibition of respiration and active transport of solutes from the sensitive cell. There is no specific site in the respiratory chain where pyocyanin can interact; but the fact that the pigment also promotes cyanide inhibition suggests that the binding site occurs before the action of cytochrome oxidase [92].

Additionally, pyocyanin alters the redox state of the cell preferentially depleting NADPH, but it can also act on glutathione, a fact that is advantageous for *P. aeruginosa*. Concentrations from 130 µM of glutathione result in the formation of H_2_O_2_, 30 times less when compared to the NADPH-Pyocyanin system [67]. In addition, glutathione inhibits the toxicity of pyocyanin in the host cell given that the molecule is an important antioxidant that can prevent the oxidative stress through the removal of ROS [104].

Most microbes, however, can synthesize some metabolites with an inhibitory action that can affect *P. aeruginosa*. These compounds are secreted to inhibit, but not to kill potential competitors [102]. Negative ecological interactions between coexisting microbial species play a crucial role and thus maintain the balance between populations in a given microsystem. In this way, none of them is expected to become dominant, avoiding the collapse of the entire trophic chain involved [116]. In this context, the antimicrobial activity of pyocyanin can also be reduced by some organisms sensitive to it, for which they experience amensalism as the most obvious response strategy [117]. Amensalism is an ecological relationship in which the production and secretion of metabolites occurs to promote inhibition of a potential competitor, without favouring the antagonistic microbe, except to remain in that environment, coexisting with its competitor [118].

The relationship between *Escherichia coli* and *P. aeruginosa* is a good example of amensalism. *E. coli* can exhibit a diversity of metabolites to restrain the physiological advantages of *P. aeruginosa*. Some of them have been exploited as in the production of indole and acetate. Especially indole in concentrations between 0.5–2.0 mM can reduce the production of pyocyanin, as well as the formation of biofilm by *P. aeruginosa* [109]. In addition, the response to oxidative stress caused by pyocyanin may result from the expression of Mn-superoxide dismutase (Mn-SDO) and other SDO [32].

Enzyme expression is the preferred mechanism of many microbes against phenazine action, for example *S. aureus* which involves peroxidases in addition to persistence such as small colony variants (SCV) [119]. *Bacillus subtilis* produces NO, stimulating the synthesis of SDO [9]. On the other hand, given the need for oxygen to enhance the effect of pyocyanin, facultative microbes and strict anaerobes may naturally be more resistant to pyocyanin [50].

## 7. Benefits of Pyocyanin for *Pseudomonas aeruginosa*

As discussed earlier, pyocyanin is not merely a secondary metabolite related to the competitive advantages of *P. aeruginosa*. The pigment is important for cellular respiration because with the increase in the concentration of pyocyanin, the assimilation of oxygen by the cell is also increased [120]. Additionally, the pigment is a physiological signal for upregulation of the genes of the QS systems during the stationary phase. This function gives the pigment a primary importance in tuning cells to a particular physiological state, transcending the simple antibiotic effect in coordinating responses by microorganisms and communities to changes in the environment [37].

Pyocyanin also participates in the release of eDNA into the environment. The origin of eDNA has been assumed to be primarily from lysed cells [121]. The release occurs through lysis promoted by the accumulation of H_2_O_2_. The release of eDNA is mediated by the QS, AHL, and PQS systems, via flagella and pili type IV. The result of this is the greater cell-cell interaction, due to changes in the properties of the cell surface, as well as in the physical-chemical interactions, important for the establishment and stability of biofilms [122]. The pyocyanin-eDNA complex interferes with the hydrophobicity of the cell, creating conditions for the formation of robust biofilms. In addition, aromatic amino acids, especially proline, histidine, arginine, leucine, tyrosine and valine, promote an up-regulatory effect on biofilm [123]. Thus, the ecological role of pyocyanin is extremely important to *P. aeruginosa* [124]. In contrast, glutathione can reverse this effect. The molecule binds directly to pyocyanin, modifying its structure and inhibiting its activity [67].

Pyocyanin is involved in metals uptake and tolerance [125]. Iron uptake by *P. aeruginosa* is also associated with pyocyanin, even under low oxygen concentrations. For Fe^3+^ to be reduced, the bacterium uses NADH and siderophores [126]. On the other hand, pyocyanin acts as a physiological signal that regulates genes involved in efflux pumps [127], giving more resistance to metals, especially silver [128].

Acting as an opportunistic pathogen, pyocyanin is relevant to inflammatory responses [129,130]. A study with wild and clinical strains found the pigment is important for the *Pseudomonas* virulence: 95% of clinical strains and 100% of wild strains produced pyocyanin. Non-producers were more sensitive to the immune system. The fact that they do not produce pyocyanin was identified with the non-expression of phzM and phzS, however, this did not prevent the bacterium from expressing other phenazines [131], as well as showing other phenotypes related to virulence [132].

Another important advantage of the expression of pyocyanin and other active phenazine, such as PCA, is the fact that oxidative stress provides a common strategy in the microbial world: overexpression of pyocyanin as a natural resource to avoid social cheating and select the most virulent strains. This event is known as social policing by co-workers, translating the concept of metabolic prudence, i.e., when the cell launches a public good preferably at no or low cost to itself. Under these conditions non-producing strains cheaters provide no benefits and are eliminated [133].

## 8. Mechanisms of Resistance of *Pseudomonas aeruginosa* to Pyocyanin

Pyocyanin is toxic, but *P. aeruginosa* has a resistance to oxidative stress resulting from pigment activity. The activation of this mechanism is associated with the concentration of intracellular phosphate. Briefly, the expression of catalase, SDO and oxidoreductases occurs, which promotes a redox cycle mediated by NADPH, neutralizing the pyocyanin from its protonated state and thereby balancing the intracellular medium, keeping the cell stable in the environment and prolonging its stationary state [134]. There is also a mechanism to avoid the toxic effects of cyanide (CN^−^). The bacterium appears to make use of active mechanisms of detoxification and synthesis of the respiratory chain, in which oxidases are insensitive to CN^−^ [135].

Secondary metabolites may also be associated with inhibition or reduction of the rate of cell growth [136]. Pyocyanin may also modulate the growth of *P. aeruginosa* in the final stages of the logarithmic phase and at the beginning of the stationary phase. This hypothesis is supported by results of previous studies [37,137,138]. It is suggested that the phenomenon is directly related to the formation of the ion transport system, as well as the generation of CN^−^, a product of glycine metabolism [139]. The role of the cyanide ion for the bacterium is not yet clear. As a virulence factor, CN^−^ is extremely toxic and binds irreversibly to the terminal of the oxidases in the respiratory chain, inhibiting aerobic respiration [140,141].

In addition, pyocyanin also promotes increased expression of genes and operons that regulate redox transport and control, as well as decrease the expression of genes and operons involved in the acquisition of Fe^3+^. In contrast, the reduction in the expression of pyocyanin also allows cells to not be physiologically disturbed [69].

## 9. Production of Pyocyanin on a Laboratory Scale

In the 1950s, many attempts were made to produce an ideal growth medium for *P. aeruginosa*, including adding glutamine to common enrichment media as a primary source of nitrogen for the synthesis of the phenazine ring. King et al. (1954) [142] proposed two semi-synthetic means to produce fluorescent pigments, verifying the inhibitory effect of phosphate. Pseudomonas A broth, known today as King A, was chosen as the standard culture medium for the detection and production of pyocyanin. King A serves as the basis for all other culture media described or modified later [143,144,145,146].

Under laboratory conditions, the amount of pyocyanin produced can vary both for the same strain or for isolated, subjected to the same incubation conditions, and on different occasions. This is possibly related to the stresses to which bacteria are exposed when kept in the laboratory [147]. Each *P. aeruginosa* strain alone has a maximum limit of pyocyanin concentration it can produce and the pigment production probably does not increase when the growth medium is modified [137]. The level of nutrients, and variation of pH, temperature and aeration are the limiting extrinsic factors for controlling the amount of pyocyanin produced [148].

The main approach in terms of the production of bacterial pigments is based on the QS strategy. Thus, commercially desired pigments can be overproduced through the induction of cellular communication when minimum nutrients required by the bacteria and high levels of autoinducers are provided [149]. This can optimize the productivity and yield of the process, as has been observed for other pigments such as prodigiosin [150] and violacein [151]. It is suggested that the presence of pyocyanin can organize the population and synchronize the expression of genes that promote the synthesis of more pyocyanin [152]. The presence of pyocyanin in the medium used during the pre-inoculum may ensure that the pigment present at the time of inoculation may induce a gradual increase in the concentration of pyocyanin.

Phosphorus as PO_4_^3−^, is the crucial nutrient for the regulation of different secondary metabolites in microorganisms; *P. aeruginosa*, especially, is very sensitive to the variation in the concentration of the phosphate ion [30]. Limiting values of phosphate imply energy reduction and pyocyanin acts as a response against nutritional stress, regulating intracellular concentration of ATP [17]. This is one of the mechanisms proposed for explaining the most important characteristics exhibited by *P. aeruginosa*: versatility, resilience and persistence in environments with high selective pressures [153].

If glycerol is included in the medium, as a non-conventional and low-cost material, rich in chorismate [154] or nitrogen [108], it can be used to obtain pyocyanin. The reuse of waste as a substrate for pigment synthesis has already been demonstrated. Oliveira et al. (2019) [155] obtained between approximately 20 and 60 μg/mL of pyocyanin, studying different concentrations of beer malt bagasse. Additionally, Cavalcanti et al. (2017) [156] observed production of pyocyanin in reactors containing peanut, cotton and sesame cakes during the removal of hydrocarbons by *P. aeruginosa* in the soil. This finding, attributed to pyocyanin, suggests a possible participation in the mechanisms used by the bacteria during the assimilation of recalcitrant compounds as sources of carbon and energy, possibly associated with the mechanisms of QS for the synthesis of biosurfactants and other tensioactive agents [147,157].

## 10. Pyocyanin Applications and Perspectives

The bioprospecting of microbes and their bioactive products is a topic of great interest and much explored, whose status has been improved since the beginning of the 21st century. Along with new compounds discovered, science has turned to the past, bringing to light some bioactive compounds discontinued for decades, yet gaining new applications [158]. Natural products and their structural analogues made up about 60% of the new small molecules that entered or returned to the market between the end of the 20th century and the beginning of this century [159]. In this context, the use of bioactive and biodegradable natural substances are more attractive strategies since they can be used to substitute synthetic compounds, related to numerous environmental impacts [160]. Table 2 lists some applications of pyocyanin in different areas of biotechnology.

The two greatest potential perspectives for the use of pyocyanin are in industry or in pharmacology, given its antimicrobial nature. The bioprospecting of pyocyanin is based on the fact that the pigment is an active redox compound, i.e., an extracellular electron carrier agent, with NADH as the initial endogenous and molecular reducer [107,128]. Microbial fuel cells (MFC) are devices that convert metabolic energy into electrical current using *P. aeruginosa* or other bacteria [170]. As a way of taking advantage of the metabolic potential of *P. aeruginosa*, the electric current can be generated using conventional substrates [171], and unconventional substrates, such as toluene, a toxic compound that is degraded by *P. aeruginosa* to produce electrical current [127]. Crude glycerol, a co-product of the process of obtaining biodiesel and the largest residue in this industrial sector, has also produced promising results [138].

Active redox compounds use the energy difference between a donor and electron acceptor, subsequently forming an ion gradient across the membrane. Electron transfer is catalysed by compounds present in the outer membrane or secreted by the cell. The addition of surfactant can increase the permeability of the membrane by up to four times. The gradient helps in the synthesis of ATP and transforms the difference in electrical potential into chemical energy. The bacterial electron flow is directed to an anode and then to an external circuit. At the cathode, electrons are used to convert oxygen into water. In addition, the pyocyanin used as an electron shuttle, increases not only the electron transfer with *P. aeruginosa*, but also with other species used in MFCs [172].

On the other hand, alterations in the respiratory chain of genetically modified *P. aeruginosa*, induced by 3,5-dichlorophenol present in water, can serve as a basis for the use of pyocyanin as a biosensor at pH = 7. The pigment can be used in its protonated state. When oxidized, the colour of the pyocyanin changed, signalling the presence of the toxin [168]. In addition, this principle can also be applied for the rapid diagnosis of *P. aeruginosa* infections [173]. Optimization of methodology could be expected to improve the biosensor’s sensitivity.

The use of pyocyanin-producing *P. aeruginosa* strains also finds frontiers in bioremediation, especially in the removal of hydrocarbons from petroleum [156]. The expression of pyocyanin may have contributed evolutionarily by the genetic adaptation of the bacteria to the degradation of the oil [174]. It should be noted that the pigment does not exhibit surfactant or emulsifying properties [147], but its participation in the assimilation of the oil seems to be involved in cellular signalling for the synthesis of these compounds. Pyocyanin can serve as an autoinducer in the expression of biosurfactants, as well as increase tolerance to toxic compounds [137]. The mechanism still needs to be understood. What is known, however, is that hydrocarbonoclastic strains of pyocyanin-producing *P. aeruginosa* synthesize more biosurfactants than those that do not exhibit the pigment [157]. This is important because oil emulsification is one of the crucial points for the assimilation of hydrocarbons as a source of carbon and energy for the bacteria [175].

On the other hand, there are numerous examples of organisms displaying amensalism through antibiosis, inspiring new defence formulations against human, animal and plant pathogens [176]. In this context, the antimicrobial activity of pyocyanin, in terms of pharmacological properties, can be exploited from two new points of view.

The first, more classic, is as an antibiotic. Good results have been achieved with successful attempts in animal models. The production of an ointment containing 5 mg/mL applied to wounds contaminated by *S. aureus*, *K. pneumoniae* and *C. albicans* promoted the elimination of these pathogens on the skin of rabbits [164]. An even more successful and safer application of pyocyanin has been as an antibiotic in aquaculture in its use against vibriosis, a disease with a high mortality rate, especially in shrimp culture. A millimolar dose already causes damage to susceptible cells, however, pyocyanin can be a safe antibiotic and used in much higher doses. The toxicity of pyocyanin is dose-dependent and can be controlled with the use of antioxidants. The application of 5–10 mg/L of pyocyanin in the treatment of vibriosis has not demonstrated a pathological effect in eukaryotes and can be used as a probiotic against the disease. In addition, in place of being effective against *Vibrio* spp., pyocyanin also exhibited activity against certain aquatic fungi [177].

The second use of the pharmacological properties of pyocyanin gives it the status of agrochemical. One of the most promising results concerns the use of the pigment in the biocontrol of fungal and bacterial phytopathogens important in different crops [10,178]. Agrochemicals are natural bioactive molecules with potential use as a pesticide. Recently, pyocyanin demonstrated activity as potentially agrochemical against *Magnaporthe grisea* and *Xanthomonas oryzae* in concentrations of 150 and 200 mg/L, respectively [166].

In the same study, as a secondary way, a new property for the pigment has been demonstrated. Applying the biocolourant concept, the researchers treated samples of white cotton fabric with a pyocyanin solution and achieved solid and durable colours, varying from lilac to pink, suggesting use of pyocyanin is an alternative source as a natural textile colour agent, endowed with strong durability and an interesting spectrum of colours [166]. This opens up a new area of research, in the context of bioprospecting for environmentally friendly materials and their derivatives.

## 11. Conclusions

In this review, we have discussed some of the recent advances in our understanding of pyocyanin by making a comprehensive assessment of its history and examining the role of the pigment to *P. aeruginosa* in terms of the bacterium relationship with the environment and other organisms, including plants, animals and microbes. Bioprospecting may uncover new strategies to take advantage of the knowledge gained from studying *P. aeruginosa* pigments aiming environmentally-friendly applications.

## Figures and Tables

**Table 1:** Summary of some susceptible organisms to pyocyanin.

Organisms	References
**Prokaryotes**	
*Bacillus* sp.	[43,105,106,107]
*Bacillus cereus*	[108]
*Bacillus megaterium*	[108]
*Enterobacter aerogenes*	[35]
*Escherichia coli*	[7,34,35,43,95,105,106,107,108,109]
*Klebsiella oxytoca*	[95]
*Proteus mirabilis*	[95,105,106]
*Pseudomonas* sp.	[34]
*Salmonella typhi*	[108]
*Shigella* sp.	[110]
*Staphylococcus aureus*	[43,95,105,106,108]
*Staphylococcus epidermidis*	[110]
*Xanthomonas oryzae*	[10]
*Vibrio* sp.	[111]
**Eukaryotes**	
*Alternaria* sp.	[110]
*Amoeba* sp.	[94]
*Aspergillus fumigatus*	[112,113]
*Aspergillus niger*	[34,43,110,113]
*Candida* spp.	[34,43,102,107,110,114]
*Candida albicans*	[112]
*Candida neoformans*	[114]
*Caenorhabditis elegans*	[115]
*Fusarium* sp.	[34]
*Penicillium* sp.	[110,113]
*Rhizoctonia solani*	[10]
*Rhizopus* sp.	[110]
*Rhodutorula* sp.	[110]
*Trichophyton* sp.	[110]

**Table 2:** Summary of some biotechnological application for pyocyanin.

Application	Reference
Clinical diagnosis	[123]
Bioremediation	[147,161]
Microbial fuel cells (MFCs)	[9,57,138,162,163]
Antibiotic	[164,165]
Agrochemical	[73,166,167]
Biosensor	[168]
Use in Organic Light Emitting Devices (OLED)	[169]
Probiotics	[111]
Antitumour	[154]
Biocolourant	[166]
